# Case Report: Iatrogenic Dental Progress of Phantom Bite Syndrome: Rare Cases With the Comorbidity of Psychosis

**DOI:** 10.3389/fpsyt.2021.701232

**Published:** 2021-07-21

**Authors:** Motoko Watanabe, Chaoli Hong, Zhenyan Liu, Chihiro Takao, Takayuki Suga, Trang Thi Huyen Tu, Tatsuya Yoshikawa, Miho Takenoshita, Yusuke Sato, Norihisa Higashihori, Keiji Moriyama, Haruhiko Motomura, Akira Toyofuku

**Affiliations:** ^1^Department of Psychosomatic Dentistry, Graduate School of Medical and Dental Sciences, Tokyo Medical and Dental University, Tokyo, Japan; ^2^Department of Basic Dental Sciences, Faculty of Odonto-Stomatology, University of Medicine and Pharmacy, Ho Chi Minh City, Vietnam; ^3^Department of Gerodontology and Oral Rehabilitation, Graduate School of Medical and Dental Sciences, Tokyo Medical and Dental University, Tokyo, Japan; ^4^Department of Maxillofacial Orthognathics, Graduate School of Medical and Dental Sciences, Tokyo Medical and Dental University, Tokyo, Japan

**Keywords:** phantom bite syndrome, occlusal dysesthesia, psychosis, schizophrenia, bipolar disorder, medical collaboration, psychiatric background, case report

## Abstract

**Introduction:** Phantom bite syndrome (PBS) is considered as the preoccupation with dental occlusion and the continual inability to adapt to changed occlusion. These patients constantly demand occlusal corrections and undergo extensive and excessive dental treatments. We present three cases with PBS-suspected iatrogenic concerns and the attribution to underlying psychosis.

**Case Presentation:** A 70-year-old female demanded orthodontic retreatment and complained of tightness and cramped sensation of teeth in the oral cavity, uncomfortable occlusion, and pain in her neck and legs that she was convinced was induced by orthodontic treatment. However, even earlier than the orthodontic treatment, she had kept doctor shopping for over 35 years, not merely dentists but also psychiatrists, neurologists, and so on; she was diagnosed with bipolar disorder. A 48-year-old female complained of malaligned improper occlusion and demanded occlusal adjustment. These symptoms occurred in the absence of a dental trigger and were worsened by orthodontic treatment. She underwent psychiatric treatment for 15 years with a diagnosis of bipolar disorder. A 38-year-old female, who had a history of schizophrenia for over 20 years, complained of occlusal discomfort and revisited with a complaint of abnormal occlusion due to excessive dental procedures. In the last two cases, requests for dental procedures had reduced owing to the collaboration between the psychiatrists and dentists. All the cases first visited our clinic following a succession of dental visits. They were strongly convinced that occlusal correction was the only solution to their symptoms, including the symptoms of discomfort in other body parts. Their misleading perceptions were uncorrectable, and repeated dental treatments exacerbated their complaints. Moreover, the dentists overlooked the psychotic histories of the patients, while the comorbid psychosis resulted in a strict demand for dental treatment by the patients.

**Conclusions:** The presented PBS cases with psychosis suggest that repeated dental treatments and comorbid psychosis exacerbate PBS. Moreover, their persistent demands reflecting comorbid psychosis led dentists to perform numerous procedures. Early detection of underlying psychosis and the prompt collaboration between psychiatrists and dentists are integral to help prevent complications in PBS cases with psychosis.

## Introduction

Phantom bite syndrome (PBS) was originally named by Marbach (1, 2), and it is also known as “occlusal dysesthesia” (3). PBS is characterized as perpetual uncomfortable occlusions that are not identifiable as dental findings nor other disorders in orofacial area. Several characteristics are indicators for the diagnosis of PBS (4): (1) repeated but unsuccessful occlusal treatment, (2) discrepancy between the subjective sensory complaints and objective occlusion, (3) belief that the occlusion only causes their medically unexplained symptoms, (4) description of their symptoms in detail, (5) laying blame on the dentists who previously treated them, and (6) high expectations for a new dental treatment.

Clinically, patients with PBS complain of an unpleasant perception of teeth contact despite repeated dental treatments, including occlusal adjustment, realignment, restoration, and change in the prosthesis. These dental treatments would be responsible for the onset of PBS triggers in some cases. The patients are preoccupied with their dental issues and seek “ideal” occlusion with a strong conviction that “bite correction” is the only means to resolve their impairment (5). The requirements placed are sometimes too intense, causing endless rounds of dentist visits and treatments. In addition, psychological and psychosocial stress is common in patients with PBS, as well as nonspecific complaints in other body parts; headache, dizziness, fatigue, misalignment of body, back pain, low back pain, pain on legs or arms, somatic or bowel problems, and so on are also observed. Comorbid psychiatric disorders were observed in 48.5–66.0% of the patients with PBS (6, 7); however, most of them were somatic symptom disorders or in the remission period of depression. Psychotic comorbidities such as schizophrenia or bipolar disorders accounted for only 5.4% of the cases. Since most patients with PBS mainly complain about their uncomfortable occlusion without severe psychiatric disorders, they would not be subjected to psychiatric treatments but head for dentists. The absence of a dental trigger at onset and the presence of a history of psychiatric disorders have been reported as predictors for a worse prognosis (7). The comorbidity of psychosis is rare; however, dentists could be faced with many difficulties in the diagnosis and treatment of such cases. If dentists underestimate comorbid psychosis and give dental procedures according to the requests of patients, the turbulent dental situations which psychiatrists can hardly notice would be induced. Here, we report PBS cases with psychosis in whom PBS was induced without any dental triggers but was exacerbated by repeated dental treatment, such as orthodontic treatments, which yielded iatrogenic concerns as a result.

## Case description

### Case 1

**Figure 1:**
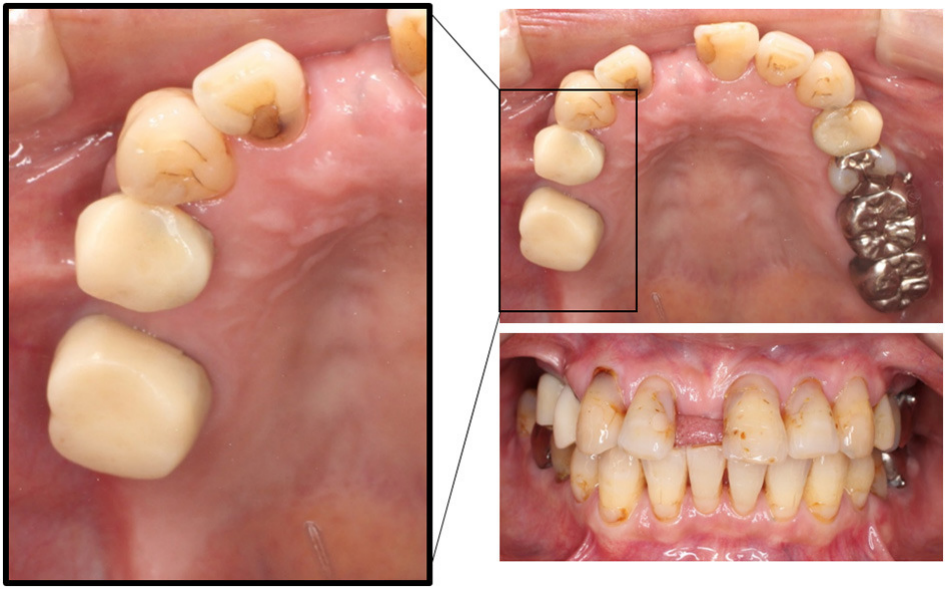
Intraoral pictures at the first visit in case 1. The bridge on her upper right molars was removed and replaced by individual temporary crowns.

A 70-year-old female, who previously worked as a Japanese teacher at a junior high school, complained that her lower left molars were moving lingually and that her oral cavity was becoming narrow. According to her assertions, her “wrong occlusion” had been causing oral dryness and burning sensation in addition to pain in her neck and legs. She recounted that these discomforts started at *X*−1 years; however, her dental and medical histories were longer and more complex. At *X*−10 years, orthodontic treatment was initiated, which was followed by her complaints of nonspecific sensations in other parts of the body such as numbness in her hand and back pain. The orthodontic treatment was successfully completed; however, her complaints worsened following the placement of a bridge on her upper right molar at *X* – 5 years. Therefore, despite no evident dental problem, the bridge was removed and replaced by individual temporary crowns owing to her persistent demands (Figure 1). She claimed that the orthodontic treatment affected her cranial nerves and caused these oral and body dysfunctions. She fervently demanded orthodontic retreatment; she was subsequently referred to our department from orthodontists at *X* years.

Her complaints seemed to have appeared following orthodontic treatment; however, a further medical interview revealed that there already had been signs of psychiatric impairment before the orthodontic treatment. She had been doctor shopping for over 35 years, which included dentists, psychiatrists, neurologists, orthopedists, dermatologists, ophthalmologists, etc., to resolve her many uncomfortable symptoms, for example, dizziness; palpitation; bloodshot eyes; pain in the eyes, nose, and throat; insomnia; vulvodynia; and so on. She had been taking ethyl loflazepate and etizolam irregularly; however, the details of her past psychotic history and treatment were unclear because she had kept wandering many psychiatrists and never managed accurately. At *X*−5 years, she was referred to another psychiatrist at our hospital from neurologists and was diagnosed as bipolar disorder with somatoform disorders or pain disorders. Regardless of the recommendation from the psychiatrist at our hospital, she had never undergone the continuous psychiatric treatment and kept doctor shopping.

At her first visit to our clinic in *X* years, although subjective oral dryness and burning sensation in her oral cavity were also complained, these oral symptoms were merely associated with her chief complain of “wrong occlusion.” Therefore, we diagnosed her symptoms as PBS and explained the typical symptoms of PBS and persuaded that orthodontic retreatment would never cure her symptoms but could probably worsen them. However, she refused our persuasions with her strong conviction that faulty occlusion was the sole reason for all her problems and demanded retreatment or referral to other dentists.

### Case 2

**Figure 2:**
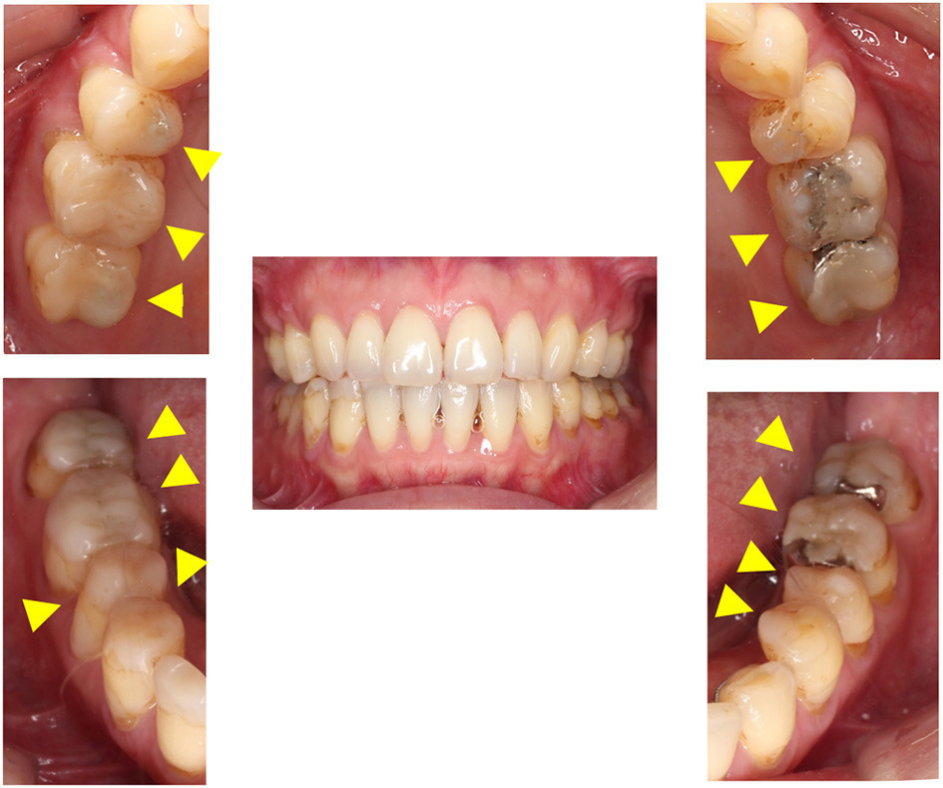
Intraoral pictures at the first visit in case 2. All teeth contacted but the dental composite resins were constructed on almost all her lower teeth for the occlusal adjustment following orthodontic treatment.

A 48-year-old female, a labor and social security attorney, complained that her occlusion was stressfully too high and sought for the alignment of her teeth since she continuously experienced suicidal thoughts owing to the uncomfortable occlusion. At *Y*−5 years, she suddenly started to feel anxious regarding the protrusion of her frontal teeth and decided to undergo orthodontic treatment. However, orthodontic treatment exacerbated her symptoms. She complained of shifting of her mandibular teeth on the left side and found it hard to locate the correct bite position. Despite repeated occlusal adjustments, the occlusion never matched the one that she desired. She recounted that it was hard to continue with her job. Almost all her lower teeth were fabricated abnormally high when she was referred to our clinic at *Y* years (Figure 2). The previous orthodontist informed us that her demands were aggressive and threatening such that they had to comply with the occlusal adjustments.

Considering her psychiatric history, at *Y*−15 years, she was found to be depressed and she showed manic episodes and was diagnosed with bipolar disorder at *Y*−11 years. She presented strong suicidal thoughts for which hospitalization had been suggested, but she always denied it citing the reason that she needed to care for her pet. The insight in bipolar disorders was lacking. She had been prescribed lamotrigine 300 mg, quetiapine 50 mg, clonazepam 0.5 mg, lorazepam 0.5 mg, and olanzapine 2.5 mg (use as needed) per day at her first visit to our clinic.

We have diagnosed her as PBS at *Y* years. The antidepressants might be effective in several patients with PBS (7, 8); however, antidepressants have a risk to make mood disorder worse. On consultation with her psychiatrist, aripiprazole 1.0 mg was added. The treatment was initiated by both psychiatrists and dentists in a cooperative manner. No unanticipated events were observed. She has been advised to refrain from further occlusal changes, and she has accordingly avoided occlusal alterations. Six months later, her demands for dental treatment have declined gradually by continuing medication and collaboration between dentists and psychiatrists.

### Case 3

**Figure 3:**
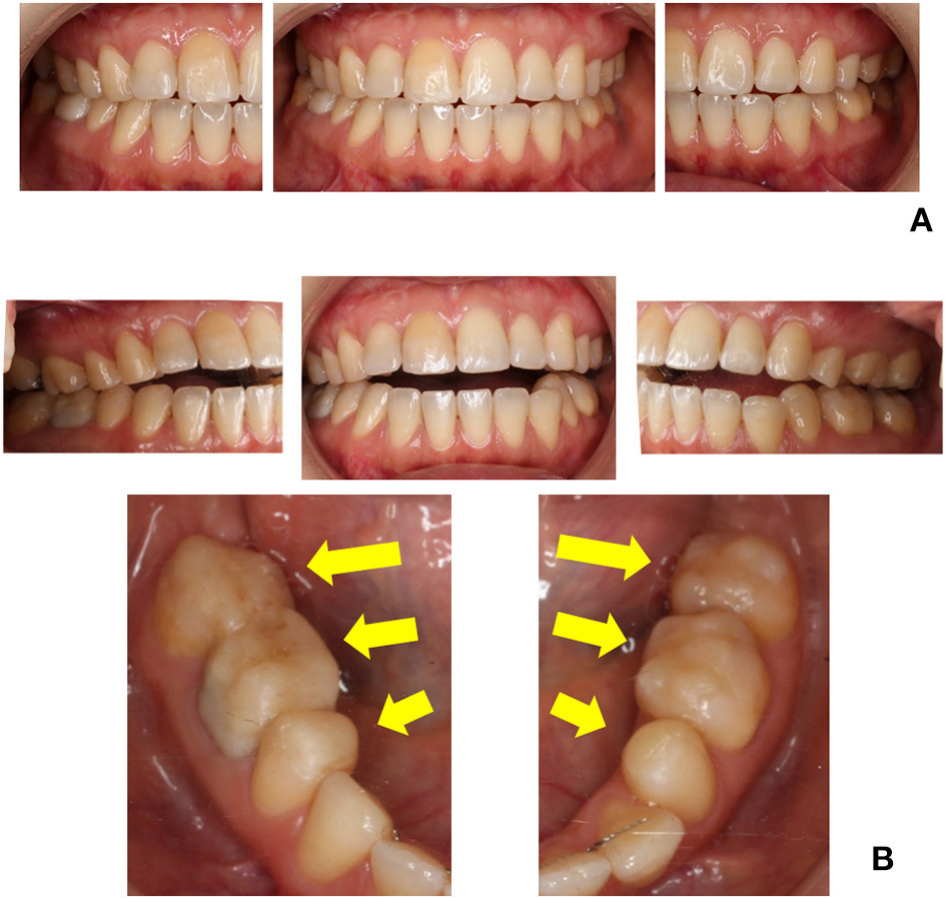
Intraoral pictures at the first visit **(A)** and revisit **(B)** in case 3. The dental composite resin was constructed on her molars according to her demands; however, anterior open bite was observed.

A 38-year-old female, a clothing sales assistant, presented with chief complaints of improper malaligned occlusion, unstable mandibular position, and neck pain. At *Z*−13 years, these symptoms occurred without any trigger. At *Z*−12 years, she underwent orthodontic treatment for her uncomfortable symptoms; however, her condition did not improve. At *Z* years, she was referred to our clinic following consultation with many dentists, including specialists in orthodontics, prosthodontics, and operative dentistry (Figure 3A). She complained one-sidedly and seemed to struggle to listen to our explanation.

Based on the note of her psychiatrist, at *Z*−21 years, she was diagnosed with neurosis following a complaint of rumbling stomach. She gradually presented social maladaptation at *Z*−13 years and was subsequently diagnosed with schizophrenia at *Z*−11 years. At *Z*−7 years, she was hospitalized for 6 months, and electroconvulsive treatment was performed. Subsequently, although anosodiaphoria was observed, neither loss of the personality level nor thought disorder was observed, and she was able to continue working. She was taking olanzapine 1.5 mg, lorazepam 0.5 mg, and Kampo medicine at *Z* years.

At *Z* + 1 year and 4 months, She revisited our clinic and more persistently demanded the addition of composite resin on her molars. The composite resin built up on the lower molars on both sides was already high enough to induce an open bite (Figure 3B). She had continued to receive occlusal adjustments every 1–2 weeks and attributed occlusion adjustment as the solution to her symptoms. She spoke continuously and never listened to our explanation of her diagnosis of PBS. The previous dentists conveyed that she returned soon after the dental treatment and always made constant phone calls. We insisted that she never undergo any further dental treatment and consulted with her psychiatrist regarding the proposal of additional treatment with aripiprazole. At *Z* + 2 years, she agreed to the prescription of aripiprazole. After the consultation with her psychiatrist, lorazepam was replaced with aripiprazole 1.0 mg, and olanzapine 2.25 mg and Kampo medicine were continued. Besides keeping patients distracted and apart from refraining from occlusal adjustment, psychopharmacotherapy without any adverse events and supportive collaboration have changed her behavior. She was able to wait for appointments and gradually calmed down; also, she made fewer demands for occlusal adjustment. Six months later, she had been prescribed aripiprazole 3.5 mg and olanzapine 2.25 mg and positively stated that her way of thinking had become clear and found it easier to concentrate on her work despite her prevailing uncomfortable occlusal perception.

## Discussion

PBS is seldom associated with severe mental disorders, and the absence of a dental trigger predicts psychiatric comorbidities (7). In all the three cases presented herein, PBS was induced without any dental trigger at the onset, but their long histories of comorbid psychosis were overlooked by most of the dentists. They had persistent wrong convictions that “bite correction” was the only solution to their terrible situation, including the unpleasant symptoms in other parts of their body. The dentists in spite of their willingness and awareness could not evade the irrational dental treatments of the patient, resulting in the abnormal occlusion in all the three cases. PBS was exacerbated by repeated dental treatments.

As one of the treatments for PBS, “nontreatment,” apart from dental treatments, is recommended to avoid unnecessary change in occlusal contacts (4). The central dominant etiologies such as brain dysfunction and neuroplasticity have been reported besides the peripheral dominant etiologies including proprioceptive alterations. Generally, educating the patient based on the possibility of the oral–neuroscience dysfunction is helpful as it helps them understand their situation. Most listen to our explanation on the etiology of PBS based on the asymmetric brain perfusion and accept psychopharmacotherapy to improve their uncomfortable perception of occlusion (7–12). However, the demands of the present cases were very aggressive to evade the unnecessary dental treatments, and their misleading perception related to occlusion and body discomfort symptoms was uncorrectable, especially following their history of frequent dental treatment. Patients with psychotic disorders display persistence in details and the preoccupation with a fixed belief even after their mental states were stabilized. These clinical features of psychotic disorders may appear in some different ways as dental problems. Primarily, hyperactivity may lead to doctor shopping, aggressiveness may be reflected by belligerent demands, and agitation may affect exacerbation. These characteristics are intertwined in a complex manner and reflected in stubborn demands, unreasonable attitude, and persistence in their own beliefs on the need for occlusal corrections.

PBS cases with such unreasonable demands have been reported, and all have been suspected of having some underlying psychiatric disorders (1, 2, 13). Psychotic disorders, however, such as schizophrenia and bipolar disorder, were rare (7, 14), while comorbid psychiatric disorders were observed in 48.5–66.0% of patients with PBS (6, 7). In the cases reported herein, long psychotic histories of over 15–35 years were prevalent in all cases: bipolar disorder in cases 1 and 2 and schizophrenia in case 3. Moreover, all the three cases showed the lack of insight regarding their psychosis. Cases 1 and 3 had a history of hospitalization, and case 2 had been recommended hospitalization many times; however, all the presented cases did not show specific features of psychosis (hallucinations, delusions, and disorganized thought processes) (15) at the initiating dental procedures. In addition, all cases were full-time employees with relatively high social adaptation and economic status. Therefore, in such cases, it is difficult for dentists to discern psychotic backgrounds; moreover, sometimes, dentists also underestimate the self-declaration of patients regarding their psychiatric history. Psychiatrists seldom comprehend the chaos abounding in dentistry as a result of such patients. One reason for the high visits to dentists may be that the patients may use uncomfortable occlusions as a way to divert their attentions from the problems in life. When the patients are no longer in the acute phase of psychosis and complain of occlusal discomfort, dentists become the main practitioners who bear the brunt of the unreasonable demands of the patients. Dentists should keep in mind that the clinical characteristics of underlying psychosis may also affect patients complaining of PBS more persistently. Hidden psychosis could be reflected by uncorrectable wrong convictions, unreasonable attitude, strict persistence, and belligerent demands. In addition, all the cases presented iatrogenic orthodontic progression that generally produces substantial occlusal changes overall. Especially for the cases with long psychotic history or young onset of psychosis and the presence of medically unexplained symptoms, dentists should be more cautious before providing orthodontic treatments.

For the management of PBS, several patients with PBS respond to psychopharmacotherapy with antidepressants (4, 7, 8); however, treatment with antidepressants for PBS would become extremely difficult when the patients have comorbid psychosis. Early detection of underlying psychiatric disorders and avoiding damage to occlusion are integral. The absence of dental triggers for PBS would predict the presence of psychiatric comorbidities (7). Dentists should not hesitate to request further psychiatric history; furthermore, they should not skip this portion of history-taking or underestimate the self-declaration of patients of the same. Once iatrogenic occlusal abnormalities are yielded, false beliefs in the patients become more uncorrectable and even psychiatrists may correlate their problems to their occlusion; thus, reliable therapeutic association becomes obscure. Prior to beginning the endless round of dental procedures, dentists should consult the psychiatrists of the patients. Moreover, not just refraining from further occlusal changes but also psychopharmacotherapy, sharing medical–dental information, and collaboration between psychiatrists and dentists were helpful in cases 2 and 3. Their demands for dental treatments declined following this. In the presented cases, the underestimation of the dentists of underlying psychosis and the little concern of psychiatrists for dental problems might have led to diagnostic delay for PBS. Especially for cases with psychosis, such as presented herein, the understanding of the psychiatrists of the turbulent dental situations and collaboration between dentists and psychiatrists are warranted.

This case presentation has a limitation about the details of psychiatric assessments and treatment including prescriptions; however, each psychiatrist diagnosed patients after prudent systemic interviews and assessments.

The presented cases demonstrate the exacerbation of PBS in a vicious cycle of repeated dental treatment and comorbid psychosis, such as schizophrenia and bipolar disorders. Stubborn demands, unreasonable attitude, and uncorrectable strong belief of occlusions, which may reflect clinical characteristics of comorbid psychosis, make it difficult for dentists to stop excessive dental treatments in such patients. Early detection of psychotic disorders and prompt collaboration between psychiatrists and dentists are integral to help such rare PBS cases with psychosis break this vicious circle.

## Data Availability Statement

The original contributions presented in the study are included in the article/supplementary material, further inquiries can be directed to the corresponding author/s.

## Ethics Statement

The studies involving human participants were reviewed and approved by Ethical Committee of Tokyo Medical and Dental University Dental Hospital. The patients/participants provided their written informed consent to participate in this study. Written informed consent was obtained from the individual(s) for the publication of any potentially identifiable images or data included in this article.

## Author Contributions

MW was involved in the treatment of the presented cases and writing of the first draft and editing the manuscript. TS, TT, CH, ZL, CT, TY, MT, YS, NH, and KM were involved in patient treatment. HM reviewed and edited the manuscript. AT treated the patients and was a major contributor in writing the manuscript. All authors have read and approved the manuscript.

## Conflict of Interest

The authors declare that the research was conducted in the absence of any commercial or financial relationships that could be construed as a potential conflict of interest.
